# Reference-free SNP calling: improved accuracy by preventing incorrect calls from repetitive genomic regions

**DOI:** 10.1186/1745-6150-7-17

**Published:** 2012-06-08

**Authors:** Jinzhuang Dou, Xiqiang Zhao, Xiaoteng Fu, Wenqian Jiao, Nannan Wang, Lingling Zhang, Xiaoli Hu, Shi Wang, Zhenmin Bao

**Affiliations:** 1 Key Laboratory of Marine Genetics and Breeding, College of Marine Life Sciences, Ocean University of China, 5 Yushan Road, Qingdao, 266003, China; 2 College of Mathematical sciences, Ocean University of China, 238 Songling Road, Qingdao, 266003, China

**Keywords:** Next-generation sequencing, single nucleotide polymorphism, genotyping, maximum likelihood, mixed Poisson/normal model

## Abstract

**Background:**

Single nucleotide polymorphisms (SNPs) are the most abundant type of genetic variation in eukaryotic genomes and have recently become the marker of choice in a wide variety of ecological and evolutionary studies. The advent of next-generation sequencing (NGS) technologies has made it possible to efficiently genotype a large number of SNPs in the non-model organisms with no or limited genomic resources. Most NGS-based genotyping methods require a reference genome to perform accurate SNP calling. Little effort, however, has yet been devoted to developing or improving algorithms for accurate SNP calling in the absence of a reference genome.

**Results:**

Here we describe an improved maximum likelihood (ML) algorithm called iML, which can achieve high genotyping accuracy for SNP calling in the non-model organisms without a reference genome. The iML algorithm incorporates the mixed Poisson/normal model to detect composite read clusters and can efficiently prevent incorrect SNP calls resulting from repetitive genomic regions. Through analysis of simulation and real sequencing datasets, we demonstrate that in comparison with ML or a threshold approach, iML can remarkably improve the accuracy of *de novo* SNP genotyping and is especially powerful for the reference-free genotyping in diploid genomes with high repeat contents.

**Conclusions:**

The iML algorithm can efficiently prevent incorrect SNP calls resulting from repetitive genomic regions, and thus outperforms the original ML algorithm by achieving much higher genotyping accuracy. Our algorithm is therefore very useful for accurate *de novo* SNP genotyping in the non-model organisms without a reference genome.

**Reviewers:**

This article was reviewed by Dr. Richard Durbin, Dr. Liliana Florea (nominated by Dr. Steven Salzberg) and Dr. Arcady Mushegian.

## Background

**Figure 1:**
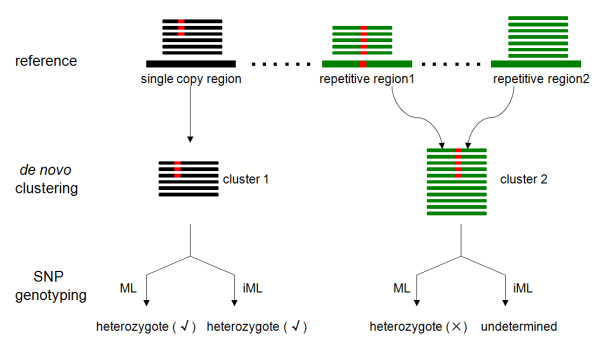
**Schematic illustration of an occurrence of a false SNP after*de novo*clustering of reads derived from repetitive genomic regions.** Both ML and iML perform well in the genotyping of SNPs derived from single-copy genomic regions (left), but iML is more efficient to identify and exclude false SNPs resulting from repetitive regions (right).

Single nucleotide polymorphisms (SNPs) are the most abundant type of genetic variation in eukaryotic genomes and have recently become the marker of choice in a wide variety of ecological and evolutionary studies such as local adaptation, population connectivity, and speciation. Many of these studies focused on non-model species, for which the number of SNPs that can be assayed are usually very limited. The advent of next-generation sequencing (NGS) technologies has made it possible to efficiently genotype a large number (e.g., thousands to tens of thousands) of SNPs in the non-model organisms with no or limited genomic resources. Several genotyping methods based on NGS platforms have recently been developed [1], most of which utilize restriction enzymes for genome complexity reduction (GCR) to reduce the total sequencing cost. In particular, RAD (restriction-site associated DNA) has gained popularity among these GCR-based methods, and allows for nearly every restriction site in the genome to be screened in parallel [2]. Most SNP calling algorithms depend on the reference-based mapping approach [3], thus limiting their use in non-model species for which a reference genome is usually not available. Little effort has yet been devoted to developing or improving algorithms for accurate SNP calling in the absence of a reference genome. Catchen et al. [4] have recently developed a pipeline program called *Stacks* for *de novo* assembly and genotyping of RAD tags from a set of individuals. The core component of their program is *ustacks*, which can efficiently build reference sites *de novo* through the assembly of short reads into clusters (i.e., stacks), and apply a maximum likelihood (ML) statistical model to distinguish SNPs from sequencing errors. Building read clusters correctly is a critical step toward accurate SNP calling, which is, however, highly sensitive to read length and genome complexity [5]. Most eukaryotic genomes contain a remarkable portion of sequences that are repetitive or close to repetitive especially on the length scale of short read. False SNPs could arise and be miscalled from read clusters in which reads carrying different sequence variants are actually derived from distinct genomic locations (i.e., repetitive regions) (Figure 1). In general, such composite read clusters should have greater depth than the normal (i.e., non-composite) ones, such that this information can be utilized to identify composite clusters and further exclude them from SNP calling. Herein, we demonstrate that the accuracy of *de novo* SNP calling can be remarkably improved using an improved ML algorithm (thereafter called iML) that incorporates the mixed Poisson/normal model to identify and exclude composite clusters from genotyping, and therefore prevents incorrect SNP calls resulting from repetitive genomic regions (Figure 1). The iML algorithm is especially powerful for accurate *de novo* SNP calling in diploid genomes with high repeat contents.

## Results and discussion

### The rationale of the iML algorithm

When a reference genome is available, the reference-based mapping approach represents an efficient way to identify and call SNPs [3]. In this approach, reads are first mapped to the reference genome, and SNPs can be identified from the sequence alignment and then genotyped by choosing one of existing SNP calling algorithms [3]. During the mapping process, reads that can be mapped to multiple genomic locations equally well are usually discarded, thus leaving no or little possibility for incorrect SNP calls resulting from repetitive genomic regions. For a RAD dataset, if the average sequencing depth is *C*, the read depth *k* for each unique restriction site theoretically follows the Poisson distribution, assuming that all sites across the genome are evenly sequenced:

(1) $$poisson\left(k|C\right)∼\frac{C^{k}e^{-C}}{k!}$$

**Figure 2:**
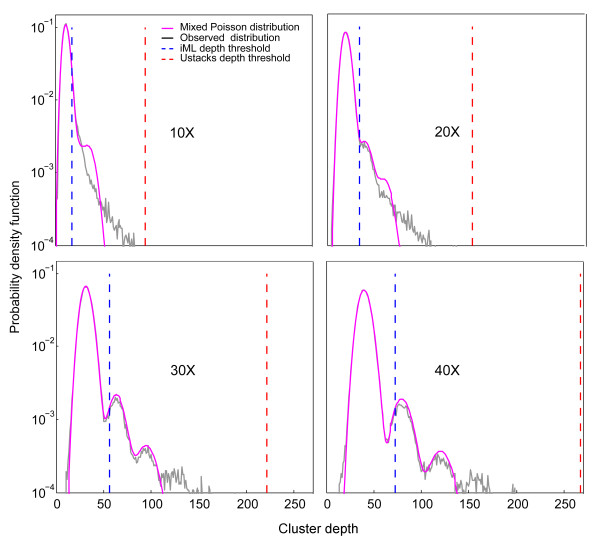
**Observed distribution of cluster depth (black) and the fitted mixed Poisson model (purple) at different sequencing depths (10x, 20x, 30x and 40x) for the simulation datasets of*Arabidopsis thaliana.*** The mixed Poisson model well fits the observed distribution especially at higher sequencing coverages. The depth threshold for genotyping is indicated by a dashed line.

However, in the absence of a reference genome, reference sites have to be established first from a large number of short reads before calling SNPs, which is usually carried out through the read-clustering approach [4]. When short reads are assembled into clusters, reads derived from repetitive genomic regions are usually unavoidably clustered together (i.e., forming composite clusters) due to high sequence similarity. In such a scenario, false SNPs could arise and be miscalled from composite clusters (Figure 1). Theoretically, the distribution of read depth of composite clusters should show a repeating pattern occurring at multiples of the average sequencing depth (*C*) (as shown in Figure 2), which corresponds to the copy number variations of repetitive sites. Therefore, in the read-clustering approach, the read depth *k* for each cluster approximately follows the mixed Poisson distribution due to the existence of composite clusters:

(2) $$\mathrm{Pr}\left(k|C\right)∼\sum_{1\leq i\leq M}a_{i}poisson\left(k|iC\right)$$

where $\sum_{1\leq i\leq M}a_{i}=1$and M indicates the copy number of repetitive sites. The mixed Poisson model is suited to describing the distribution of cluster depth derived from diploid genomes with high repeat contents, and providing the information necessary to identify and exclude composite clusters from SNP genotyping. All parameters including *C* and *a*_*1*_ ~ *a*_*M*_ can be directly estimated from the sequencing dataset using the expectation-maximization (EM) algorithm (see Methods).

The original ML genotyping algorithm was developed by Hohenlohe et al. [6] for SNP calling from RAD tags. This algorithm calculates the likelihood for each possible genotype at a given locus, and selects the one with the largest likelihood. Here we describe an improved ML algorithm called iML that incorporates the mixed Poisson/normal model and thus can exclude repetitive loci from genotyping. The posterior probabilities for each of the three possible categories (homozygote, heterozygote and undetermined) are calculated as follows:

(3) $$\begin{array}{l} \mathrm{Pr}\left(n_{1},n_{2},n_{3},n_{4}|homozygote\right)=\frac{n!}{n_{1}!n_{2}!n_{3}!n_{4}!}poisson\left(n|C\right){\left(1-\frac{3e}{4}\right)}^{n_{1}}{\left(\frac{3e}{4}\right)}^{n_{2}+n_{3}+n_{4}}\\ \mathrm{Pr}\left(n_{1},n_{2},n_{3},n_{4}|heterozygote\right)=\frac{n!}{n_{1}!n_{2}!n_{3}!n_{4}!}poisson\left(n|C\right){\left(0.5-\frac{e}{4}\right)}^{n_{1}+n_{2}}{\left(\frac{3e}{4}\right)}^{n_{3}+n_{4}}\\ \mathrm{Pr}\left(n_{1},n_{2},n_{3},n_{4}|undetermined\right)=\sum_{k\geq 2}\frac{a_{k}}{1-a_{1}}poisson\left(n|kC\right) \end{array}$$

where *n*_*1*_*n*_*2*_*n*_*3*_ and *n*_*4*_ are the read counts for each of the four possible nucleotides (A, T, C and G); n is the total number of reads and *ϵ* is the sequencing error rate. According to the formula 3, each locus is genotyped by assigning it into the category with the largest posterior probability.

### Testing the performance of the iML algorithm on simulation datasets

To test the performance of the iML algorithm, we created two series of RAD simulation datasets for *de novo* SNP calling; one was based on the model plant species *Arabidopsis thaliana* genome (~157 Mbp) [7] and the other on the relatively large rice (*Oryza sativa*) genome (~385 Mbp), which has a high repeat content (>35%) [8]. The simulation details were described in Methods. Briefly, simulation datasets were composed of *in silico* short reads (i.e., RAD tags) that were extracted from all *Eco*RI restriction sites in the genome (73,624 sites in *A. thaliana*, and 175,460 sites in *O. sativa*) at different read lengths (35, 50 and 100 bp) and different sequencing depths (from 8x to 40x). SNPs were introduced at a rate of 0.5% accompanying with 1% global sequencing error. For *de novo* read clustering, *ustacks* first assembles all reads into exactly matching clusters (i.e. representing individual alleles), and then “allele” clusters are further merged into “locus” clusters with extremely deep clusters (i.e. more than two standard deviations above the mean depth) excluded from subsequent analysis [4]. SNPs were genotyped from the obtained clusters using iML, ML or a threshold approach (minor allele frequency > 35%). The false positive rate (FPR) and false negative rate (FNR) were calculated to evaluate the performance of iML in comparison with the others. The parameters *C* and *a*_*1*_ ~ *a*_*3*_ were estimated using the EM algorithm (Additional file 1: Table S1). The mixed Poisson model well fit the simulation data, especially at higher sequencing coverages (Figure 2). Parameter 1-*a*_*1*_ accurately predicted the percentage of composite clusters resulting from *de novo* read clustering (Additional file 2: Table S2). Note, although *ustacks* has an implemented algorithm that intends to remove repetitive loci by excluding clusters that are two standard deviations above the mean depth of all clusters, this approach is much less efficient at identifying repetitive loci than ours (see Figure 2), which is largely due to the fact that some extremely deep clusters (e.g. ~0.3% of total clusters with a depth of more than 1,000 reads at the 40x sequencing coverage) can result in a very large standard deviation of cluster depth.

**Figure 3:**
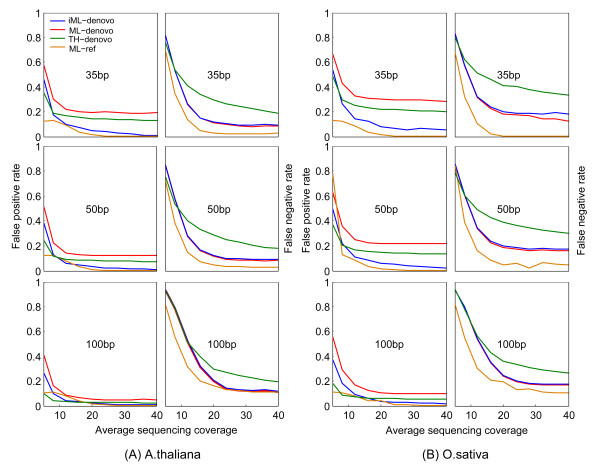
**Comparison of the performance of three*de novo*SNP calling approaches based on the simulation datasets of*Arabidopsis thaliana*(A) and*Oryza sativa*(B).** iML outperforms ML or a threshold approach by improving genotyping accuracy remarkably at the expense of little decreased sensitivity. ML_ref, reference-based SNP calling using the ML algorithm; iML_denovo, *de novo* SNP calling using the iML algorithm; ML_denovo, *de novo* SNP calling using the ML algorithm; TH_denovo, *de novo* SNP calling using the threshold approach.

For *A. thaliana*, iML always generated lower FPRs than the threshold approach or ML with 12 ~ 19%, 6 ~ 11% and 2 ~ 4% FPR reductions corresponding to the read lengths of 35, 50 and 100 bp, respectively, at a 40x sequencing depth, whereas iML generated only slightly higher FNRs (~1%) in comparison with ML (Figure 3A, Additional file 3: Table S3). For the relatively large rice genome, which has a high repeat content, the performance of iML is even more pronounced with 15 ~ 23%, 11 ~ 20% and 3 ~ 8% FPR reductions corresponding to the read lengths of 35, 50 and 100 bp, respectively, at a 40x sequencing depth but less noticeable FNR reductions in comparison with ML (Figure 3B, Additional file 3: Table S3). The threshold approach performed better than ML in terms of FPR reduction, but this was achieved at the expense of substantially decreased sensitivity (e.g. 11% FNR increase for *A. thaliana* and 21% for *O. sativa* at a 40x sequencing depth for 35-bp reads). In all cases, iML improved the accuracy of *de novo* SNP calling, bringing the accuracy close to the level resulting from the reference-based mapping approach.

### Testing the performance of the iML algorithm on real datasets

We further evaluated the performance of the iML algorithm on two real sequencing datasets. One dataset was generated by Wang et al. [9] for *A. thaliana* using a newly developed 2b-RAD method based on type IIB restriction enzymes, whereas the other was generated by Etter et al. [10] for stickleback (*Gasterosteus aculeatus*) using the standard RAD method. For the *A. thaliana* dataset, ~ 5.8 million high-quality reads were obtained, of which 91.3% could be mapped to the reference genome and 99.4% were present in *de novo* read clusters, whereas ~ 4.7 million high-quality reads were obtained from the *G. aculeatus* dataset, of which 88.6% could be mapped to the reference genome and 90.3% were present in *de novo* read clusters (Table 1). For both datasets, the number of read clusters was comparable to the number of unique restriction sites predicted from each genome (Table 1). Note, for *G. aculeatus*, the number of predicted unique sites was slightly lower than that of read clusters possibly due to the fact that the stickleback genome assembly (BROADS1) is still incomplete.

**Table 1:** Summary of two real sequencing datasets used for evaluation of the iML algorithm

	***Arabidopsis thaliana***	***Gasterosteas aculeatus***
Library preparation	2b-RAD	RAD
Restriction enzyme	BsaXI (ACN_5_CTCC)	SbfI (CCTGCAGG)
Trimmed read length	27 bp	55 bp
High-quality reads	5,845,509	4,672,098
Mapped reads	5,339,662	4,139,761
Clustered reads	5,809,558	4,220,881
No. of *in silico* restriction sites^a^	39,678	45,600
No. of *in silico* unique sites	35,362	40,125
No. of read clusters	33,877	42,352
Reference	[9]	[10]

^a^, the total number of restriction sites that were predicted from the genome assemblies of TAIR8 and BROADS1 for *A. thaliana* and *G. aculeatus*, respectively.

**Figure 4:**
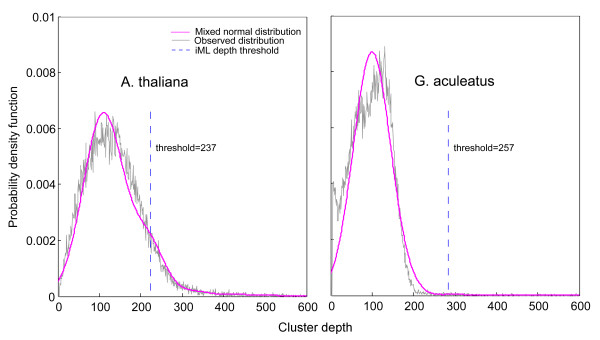
**Observed distribution of cluster depth (black) and the fitted mixed normal model (purple) for the real sequencing datasets of*Arabidopsis thaliana*and*Gasterosteus aculeatus.*** The depth threshold for iML genotyping is indicated by a dashed line

In data simulation, we assume that read depth for each restriction site follows the Poisson distribution, which is, however, may not be fully valid for real datasets due to a few practical reasons such as uneven cutting efficiency across restriction sites, amplification bias, and sequencing artifact/error. Before implementing the iML algorithm, we first performed a model fitness test for four distribution models (Poisson, mixed Poisson, normal, and mixed normal) on the two real datasets (Table 2). It turned out that the mixed normal model best fit the observed distribution of cluster depth in both datasets (Table 2, Figure 4), suggesting that unlike in the simulation analysis, the mixed Poisson model may not be the model of choice for real datasets in practice. Therefore, the mixed normal model was selected to implement the iML algorithm on the real sequencing datasets.

**Table 2:** The K-S test for the model fitness of four distribution models on two real datasets

**Dataset**	**Model**	**Estimated parameters**							***P*value**
		***C***	***a***_***1***_	***a***_***2***_	***a***_***3***_	***σ***_***1***_	***σ***_***2***_	***σ***_***3***_	
*A. thaliana*	Poisson	137.2	-	-	-	-	-	-	0
	Mixed Poisson	125.0	0.83	0.09	0.09	-	-	-	0
	Normal	137.2	-	-	-	74.1	-	-	2.5E-7
	Mixed normal	110.0	0.80	0.18	0.01	48.2	39.1	34.2	0.378
*G. aculeatus*	Poisson	98.1	-	-	-	-	-	-	0
	Mixed Poisson	105.0	0.84	0.09	0.07	-	-	-	0
	Normal	98.1	-	-	-	50.5	-	-	0.029
	Mixed normal	100.0	0.98	0.02	0.00	45.3	24.3	24.1	0.288

*C*, *a*_*i*_ and *σ*_*i*_ represent the mean, the coefficient and standard deviation of *i*-copy clusters in a given model.

**Figure 5:**
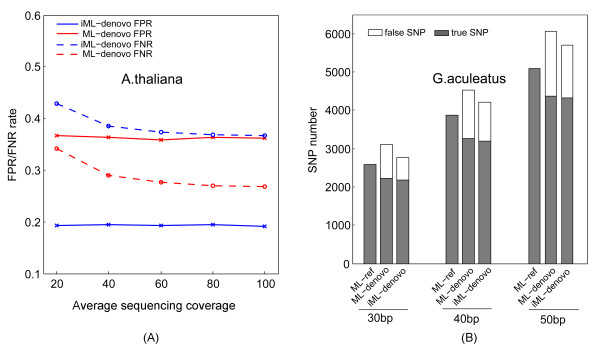
**Comparison of the performance of*de novo*SNP calling approaches based on the real sequencing datasets of*Arabidopsis thaliana*(A) and*Gasterosteus aculeatus*(B).** FPR/FNR, false positive or negative rate.

As expected, iML still generated lower FPRs than ML with approximately 17% FPR reduction at different sequencing depths for *A. thaliana* (Figure 5A), and 4% (50 bp) ~7% (30 bp) FPR reduction at different read lengths for *G. aculeatus* (Figure 5B). The performance of iML was less pronounced on the *G. aculeatus* dataset because this dataset contained much less repetitive restriction sites than the *A. thaliana* dataset (Figure 4). In comparison with the simulation analysis, iML coupled with the mixed normal model is relatively less efficient at distinguishing composite clusters from unique ones on the real sequencing data, as reflected by the observation of substantially high FPR and FNR remained in real datasets even at the deep sequencing coverages (Figure 5A). Nevertheless, iML still outperformed the original ML algorithm in terms of genotyping accuracy on the real sequencing datasets, and therefore represents the most promising algorithm currently available for accurate *de novo* SNP genotyping in diploid genomes with high repeat contents.

## Conclusions

In summary, we describe an improved ML algorithm that incorporates the mixed Poisson/normal model and can efficiently remove incorrect SNP calls resulting from repetitive regions. Through analysis of simulation and real datasets, we demonstrate that iML improved the accuracy of *de novo* SNP calling remarkably in comparison with ML or a threshold approach. The iML algorithm is especially powerful for accurate *de novo* SNP calling in diploid genomes with high repeat contents. The Perl script used for implementation of the iML algorithm is available from the authors upon request.

## Methods

### Evaluation of the iML algorithm on simulation and real datasets

Two series of simulation datasets were created for *in silico* RAD genotyping, one from the model plant species *A. thaliana* genome (~157 Mbp) and the other from the relatively large rice (*Oryza sativa*) genome (~385 Mbp), which has a high repeat content (>35%). Simulation datasets were composed of *in silico* short reads (i.e., RAD tags) extracted from all *Eco*RI restriction sites in the genome (73,624 sites in *A. thaliana*, and 175,460 sites in *O. sativa*) at different read lengths (35, 50 and 100 bp) and different sequencing depths (from 8x to 40x). SNPs were introduced at a rate of 0.5% by adding alleles to the diploidized genomes. Each allele was “sequenced” to a depth determined by a draw from the Poisson distribution at different mean sequencing depth. For each “sequenced” read, the global error rate, which increases linearly along the sequence, was set to 1%. Each simulation was replicated 10 times. The reference-based mapping approach was carried out using the SOAP2 program (parameters -M 4, -v 2, -p 1) [11], and SNPs were called using the ML algorithm. For *de novo* read clustering, *ustacks* first assembled all reads into exactly matching clusters but excluded clusters that contained less than 2 reads (parameter -m 2) because these clusters are considered to be indistinguishable from the ones generated with sequencing errors [4]. The established clusters were further merged iteratively by allowing two-nucleotide distance between clusters (parameter -M 2). The extremely deep clusters (i.e. more than two standard deviations above the mean depth) were excluded from subsequent analysis [4]. SNPs were genotyped from the obtained clusters using iML, ML or a threshold approach. In the threshold approach, only read clusters with minor allele frequency > 35% were qualified for SNP calling, and this criterion has recently been shown to perform better than the ML algorithm in terms of genotyping accuracy [9]. The parameters *C* and *a*_*1*_ ~ *a*_*3*_ were estimated using the EM algorithm (*e* = 0.000001). Note, for the sake of simplicity, only *a*_*1*_ ~ *a*_*3*_ were considered in the simulation analyses. Bootstrap analysis of the EM estimation was performed 100 times. The false positive rate (FPR) and false negative rate (FNR) were calculated to evaluate the performance of the iML algorithm in comparison with other genotyping approaches.

In order to evaluate the performance of the iML algorithm on real datasets, two RAD-related sequencing datasets were retrieved from the NCBI SRA database (SRA accession no. SRP008452 and SRX028651). One was generated by Wang et al. [9] for *A. thaliana* using a newly developed 2b-RAD technique, while the other was generated by Etter et al. [10] for stickleback (*Gasterosteus aculeatus*) using the standard RAD technique. Note, although the stickleback dataset was generated by a 2x60 paired-end sequencing, for simplification, only single-end reads that contained the restriction site were used for evaluation of the iML algorithm. The low-quality reads containing ambiguous basecalls (N), or > 5 positions with low quality score (< 10), or no restriction site, as well as reads mapped to organelles (mitochondrion and/or chloroplast) were excluded from further analysis. The *de novo* and reference-based genotyping approaches followed the same procedure as described for the simulation analysis. The Kolmogorov-Smirnov test was used to determine the fitness of four distribution models (Poisson, mixed Poisson, normal, and mixed normal) to the observed distribution of cluster depth in the real datasets. Since SNP configuration is unknown for the real datasets, we considered SNPs genotyped by the reference-based approach as “true” SNPs, which then served as the positive control for calculating the FPRs and FNRs of ML or iML algorithm.

### Parameter estimation of the mixed Poisson model

Parameters of the mixed Poisson/normal model were estimated using the EM approach. Here we describe the procedure of parameter estimation for the mixed Poisson model, which can be extended to the mixed normal model.

In the *de novo* read-clustering approach, the read depth *k* for each cluster approximately follows the mixed Poisson distribution due to the existence of composite clusters:

(4) $$\mathrm{Pr}\left(k|C\right)\sim \sum_{1\leq i\leq M}a_{i}poission\left(k|iC\right)$$

Let *n* be the observed cluster number after read clustering and *d*_*i*_ denotes the depth of the *i*th cluster. The log-likelihood of observed data has the following form:

(5) $$L=\sum_{i=1}^{n}\log\left\{\sum_{j=1}^{M}a_{j}poission\left(d_{i}|Cj\right)\right\}$$

The parameter estimates that maximize this log-likelihood are asymptotically efficient estimates of the parameters *C* and *a*_*j*_. However, the direct maximization of the log-likelihood is very difficult. Therefore, *C* and *a*_*j*_ can be estimated by the EM algorithm through successive maximizations of the expected value of the more tractable complete-data log-likelihood:

(6) $$L=\sum_{i=1}^{n}\sum_{j=1}^{M}\log\left\{a_{j}poission\left(d_{i}|Cj\right)\right\}$$

The initial values of M + 1 parameters can be defined as

(7) $$a_{1}^{\left(0\right)},a_{2}^{\left(0\right)},\cdots a_{M}^{\left(0\right)},C^{\left(0\right)}$$

The M + 1 parameters are updated as

(8) $$\begin{array}{l} a_{j}^{\left(k+1\right)}=\frac{1}{n}\sum_{i=1}^{n}P_{j}\left(d_{t},C^{\left(k\right)},a^{\left(k\right)}\right)\\ C^{\left(k+1\right)}=\frac{\sum_{t=1}^{n}d_{t}P_{1}\left(d_{t},C^{\left(k\right)},a^{\left(k\right)}\right)}{\sum_{t=1}^{n}P_{1}\left(d_{t},C^{\left(k\right)},a^{\left(k\right)}\right)} \end{array}$$

where $P_{j}\left(d_{t},C^{\left(k\right)},a^{\left(k\right)}\right)=\frac{a_{j}^{\left(k\right)}poission\left(d=d_{t}|jC^{\left(k\right)}\right)}{\sum_{j=1}^{M}a_{j}^{\left(k\right)}poission\left(d=d_{t}|jC^{\left(k\right)}\right)}$

The EM algorithm continues until $||a_{j}^{\left(k+1\right)}-a_{j}^{\left(k\right)}||\leq e,||C^{\left(k+1\right)}-C^{\left(k\right)}||\leq e$.

Let D = {d_1_,d_2_…d_n_} be the set of observed cluster depth. Bootstrap samples were created by sampling with replacement of *n* individual observations. Balance bootstrapping was performed 100 times for each sample by drawing *n* observations out of the pool of original observations. The means and standard deviations were calculated to evaluate the robustness of the EM approximation.

## Abbreviations

NGS, next-generation sequencing; SNP, single nucleotide polymorphism; GCR, genome complexity reduction; RAD, restriction-site associated DNA; EM, expectation-maximization; ML, maximum likelihood; FPR, false positive rate; FNR, false negative rate.

## Competing interests

The authors declare that they have no competing interests.

## Authors' contributions

SW and JD conceived and designed the study. JD, XZ, XF, WJ and NW conducted bioinformatic analyses. JD, LZ, XH, SW and ZB drafted the manuscript.

## Reviewers' comments

*Reviewer's report 1*

*Dr. Richard Durbin, The Sanger Institute, UK*

This reviewer provided no comments for publication.

*Reviewer's report 2*

*Dr. Liliana Florea (nominated by Dr. Steven Salzberg), Johns Hopkins University School of Medicine, USA*

The authors report on an improved ML-based method, called MP-ML, to prevent false SNP calls due to the collapse of reads from repetitive regions. The key innovation is the use of a mixed Poisson model of read coverage to identify composite read clusters, which are then excluded from subsequent analysis. The method is particularly useful for SNP calling in non-model organisms, for which there is no reference genome to help disambiguate the location of repetitive reads.

Intuitively, composite clusters will consist of reads from multiple loci with similar underlying sequence, with each locus following a Poisson distribution with the same mean read coverage C. MP-ML is shown to compare favorably to a simpler ML approach reported earlier, significantly reducing the false discovery rate (especially for short reads) with little change in sensitivity.

Unfortunately, the evaluation is done exclusively on artificial data, simulated from the A. thaliana and O. sativa genomes using precisely the Poisson model of coverage, and does not capture the complexities of real reads. Two important assumptions are that: i) all the reads at the different loci will be clustered together, and ii) there is no bias in the Poisson distribution across the genomes, either one of which may be violated with real data. For the latter, there are known systematic biases in coverage across a genome, for instance at GC-rich regions that are more difficult to sequence. To have a realistic estimate of the method's performance and hurdles in practical situations, it is essential to include some tests on real data in the assessment.

**Authors' response:** Evaluation of the performance of our algorithm (now called iML) on two real sequencing datasets has been added in the revised manuscript. Indeed, the mixed Poisson model did not fit the real datasets very well. However, we showed that the distribution of cluster depth in the real datasets could be fitted with the mixed normal model (Table 2, Figure 4). As expected, our algorithm based on the mixed normal model still outperformed the original ML algorithm in terms of FPR reduction (Figure 5A,B). In addition, our results showed that in comparison with the simulation analysis, iML coupled with the mixed normal model was relatively less efficient at distinguishing composite clusters from unique ones on the real sequencing data.

Second, the evaluation is limited to the comparison with only one tool, the ML method in (Hohenlohe et al., PLoS Genet, 2010), which also served as the starting point for developing MP-ML. Other approaches should be included and discussed, for instance the DIAL method in (Ratan et al., BMC Bioinformatics, 2010).

**Authors' response:** DIAL is a pipeline program that integrates multiple data processing steps, many of which can affect final genotyping results, therefore limiting its use for a fair comparison between different *de novo* genotyping algorithms. In essence, DIAL utilizes a threshold approach (i.e. a fixed threshold for minor allele frequency) for *de novo* SNP genotyping. Comparison between our algorithm and the threshold approach has now been added in the revised manuscript (see Figure 3, Additional file 3: Table S3).

Third, it would be very useful to present details of the algorithms in the read clustering method (Stacks), since they determine what kinds of clusters are being analyzed.

**Authors' response:** We have provided a more detailed description of the read clustering approach used in this study (see Methods). We would also like to refer readers to the ref. 4 for further details about the read clustering algorithm implemented in Stacks.

The article is very well written and organized.

Minor:

1. In the Methods, Poisson is misspelled "poission".

**Authors' response:** Corrected.

*2. In Figure*3*, the colors representing 'MP-ML de novo' and 'ML-ref' are hard to distinguish in the panels.*

**Authors' response:** This figure has been remade.

*Reviewer's report 3*

*Dr. Arcady Mushegian, Stowers Institute for Medical Research, USA*

This reviewer provided no comments for publication.

## Open peer review

Reviewed by Dr. Richard Durbin, Dr. Liliana Florea (nominated by Dr. Steven Salzberg) and Dr. Arcady Mushegian. For the full reviews, please go to the Reviewers' comments section.

## Supplementary Material

Additional file 1 **Table S1.** Estimation of parameters *C* and *a*_*1*_ ~ *a*_*3*_ for the mixed Poisson model using the expectation-maximization (EM) algorithm. Click here for file

Additional file 2 **Table S2.** Comparison of the observed and estimated percentage of composite and non-composite clusters at different sequencing depths. Click here for file

Additional file 3 **Table S3.** False positive rates and false negative rates calculated for four SNP calling approaches based on the simulation datasets of *Arabidopsis thaliana* and *Oryza sativa*. Click here for file
