# Supplementary material for: Reference-free SNP calling: improved accuracy by preventing incorrect calls from repetitive genomic regions
Source: Biol Direct. 2012 Jun 8;7:17. doi: 10.1186/1745-6150-7-17 (PMC3472322; doi:10.1186/1745-6150-7-17)
Supplement: Additional file 1 — Table S1. Estimation of parameters C and a1 ~ a3 for the mixed Poisson model using the expectation-maximization (EM) algorithm. [file 1745-6150-7-17-S1.pdf]

Table S1. Estimation of parameters C and a_1~a_3 in the mixed Poisson model using the expectation-maximization (EM) algorithm.

Dataset	Cov[a]	35 bp				50 bp				100 bp			
		C ± SD[b]	a_1 ± SD ($x10^{-2}$)	a_2 ± SD ($x10^{-2}$)	a_3 ± SD ($x10^{-2}$)	C ± SD	a_1 ± SD ($x10^{-2}$)	a_2 ± SD ($x10^{-2}$)	a_3 ± SD ($x10^{-2}$)	C ± SD	a_1 ± SD ($x10^{-2}$)	a_2 ± SD ($x10^{-2}$)	a_3 ± SD ($x10^{-2}$)
	4	5.0±0.00	94.23±0.42	2.53±0.82	3.24±0.37	5.10±0.00	94.34±0.57	1.63±0.76	4.03±0.39	5.4±0.30	89.37±1.88	5.64±1.87	5.00±0.87
	8	8.2±0.00	93.61±0.40	4.50±0.50	1.89±0.18	8.20±0.20	93.97±1.96	4.49±0.94	1.54±0.19	9.2±0.20	94.72±1.41	3.92±1.42	1.36±0.23
	12	12.1±0.50	94.39±1.20	4.12±1.02	1.49±0.24	12.1±0.22	95.21±0.82	3.77±0.79	1.03±0.13	13.1±0.10	95.71±0.35	3.31±0.35	0.98±0.11
	16	16.2±0.47	94.48±0.62	4.21±0.51	1.31±0.17	16.2±0.20	94.51±0.28	4.36±0.25	1.13±0.08	15.1±0.35	95.56±0.37	3.66±0.34	0.78±0.08
A. thaliana	20	20.1±0.49	94.58±0.40	4.36±0.37	1.06±0.11	19.8±0.39	95.74±0.33	3.48±0.31	0.78±0.06	20.1±0.29	96.90±0.24	2.51±0.22	0.59±0.07
(Wall Cress)	24	23.9±0.46	94.20±0.36	4.70±0.35	1.10±0.11	23.7±0.14	95.25±0.19	3.90±0.19	0.85±0.06	24.5±0.60	97.17±0.24	2.26±0.23	0.57±0.05
	28	27.9±0.97	94.62±0.54	4.41±0.51	0.97±0.07	27.9±0.46	95.88±0.27	3.36±0.26	0.76±0.05	28.0±0.66	97.07±0.20	2.24±0.20	0.69±0.05
	32	31.8±0.44	94.80±0.21	4.23±0.20	0.98±0.07	32.2±0.46	96.01±0.22	3.19±0.21	0.80±0.06	31.1±0.46	96.81±0.18	2.49±0.17	0.70±0.07
	36	35.9±0.47	94.66±0.23	4.23±0.23	1.12±0.07	36.0±0.62	95.63±0.21	3.52±0.21	0.85±0.06	35.4±0.41	96.68±0.18	2.52±015	0.80±0.08
	40	40.1±0.67	94.70±0.19	4.29±0.19	1.01±0.06	39.9±0.53	95.77±0.22	3.33±0.06	0.90±0.07	39.2±0.44	96.83±0.15	2.38±0.13	0.79±0.09
	4	5.1±0.00	89.33±0.42	4.64±0.55	6.03±0.26	5.2±0.00	87.30±0.55	5.69±0.71	7.01±0.30	5.1±0.00	82.56±1.10	8.07±1.15	9.37±0.51
	8	8.3±0.00	88.86±0.24	7.68±0.28	3.46±0.14	8.1±0.00	88.83±0.29	7.84±0.32	3.33±0.13	9.3±0.26	90.68±3.79	6.34±3.87	2.98±0.18
	12	12.5±0.00	90.25±0.17	7.07±0.18	2.68±0.11	12.4±0.34	91.56±0.17	6.11±0.16	2.33±0.19	12.4±0.35	91.90±0.59	6.24±0.61	1.86±0.10
	16	16.1±0.47	90.80±0.85	6.87±0.70	2.32±0.18	15.2±0.14	90.71±0.28	7.09±0.24	2.20±0.11	16.2±0.31	94.28±0.46	4.41±0.39	1.31±0.09
O. sativa	20	20.2±0.35	90.54±0.37	7.29±0.30	2.17±0.11	19.2±0.50	91.24±0.48	6.78±0.39	1.98±0.14	20.1±0.41	94.80±0.35	4.06±0.29	1.13±0.06
(Rice)	24	24.2±0.37	90.73±0.26	7.30±0.22	1.97±0.10	24.1±0.49	92.40±0.39	5.85±0.36	1.75±0.09	23.1±0.27	94.46±0.19	4.38±0.17	1.16±0.06
	28	28.1±0.52	90.67±0.19	7.36±0.16	1.97±0.18	27.3±0.36	91.86±0.21	6.39±0.17	1.75±0.09	27.2±0.50	94.60±0.28	4.36±0.26	1.04±0.05
	32	31.0±0.59	90.45±0.35	7.54±0.29	2.01±0.10	31.0±0.52	92.05±0.13	6.31±0.11	1.64±0.08	32.5±0.68	95.15±0.22	3.91±0.19	0.94±0.05
	36	36.2±0.50	90.48±0.21	7.51±0.17	2.01±0.08	36.0±0.47	92.22±0.21	6.18±0.18	1.61±0.07	35.0±0.42	94.81±0.20	4.28±0.18	0.92±0.04
	40	40.1±0.60	90.58±0.22	8.41±0.20	1.01±0.08	39.0±0.56	92.19±0.19	6.30±0.17	1.51±0.06	38.3±0.65	94.81±0.18	4.24±0.17	0.95±0.05

[a] Coverage of the simulated sequencing.

[b] Means and standard deviations (SD) were calculated from 1000 simulated analyses (10 replicates × 100 bootstrap samples) to evaluate the robustness of the EM approximation.
