# Supplementary material for: Reference-free SNP calling: improved accuracy by preventing incorrect calls from repetitive genomic regions
Source: Biol Direct. 2012 Jun 8;7:17. doi: 10.1186/1745-6150-7-17 (PMC3472322; doi:10.1186/1745-6150-7-17)
Supplement: Additional file 2 — Table S2. Comparison of the observed and estimated percentage of composite and non-composite clusters at different sequencing depths. [file 1745-6150-7-17-S2.pdf]

Table S2. Comparison of the observed and estimated percentage of composite and non-composite clusters at different sequencing depths.

Dataset	Cov	35 bp				50 bp				100 bp			
		Non-composite cluster		Composite cluster		Non-composite cluster		Composite cluster		Non-composite cluster		Composite cluster	
		Observed[a]	Estimated[b]	Observed	Estimated	Observed	Estimated	Observed	Estimated	Observed	Estimated	Observed	Estimated
	4	0.942	0.900	0.058	0.100	0.943	0.906	0.057	0.094	0.894	0.917	0.106	0.083
	8	0.936	0.940	0.064	0.060	0.940	0.949	0.060	0.051	0.947	0.948	0.053	0.052
	12	0.944	0.948	0.056	0.052	0.952	0.959	0.048	0.041	0.957	0.965	0.043	0.035
	16	0.945	0.949	0.055	0.051	0.945	0.960	0.055	0.040	0.956	0.974	0.044	0.026
A. thaliana	20	0.946	0.949	0.054	0.051	0.957	0.961	0.043	0.039	0.969	0.976	0.031	0.024
(Wall Cress)	24	0.942	0.949	0.058	0.051	0.953	0.960	0.048	0.040	0.972	0.977	0.028	0.023
	28	0.946	0.948	0.054	0.052	0.959	0.960	0.041	0.040	0.971	0.978	0.029	0.022
	32	0.948	0.947	0.052	0.053	0.960	0.959	0.040	0.041	0.968	0.978	0.032	0.022
	36	0.947	0.947	0.053	0.053	0.956	0.959	0.044	0.041	0.967	0.978	0.033	0.022
	40	0.947	0.947	0.053	0.053	0.958	0.959	0.042	0.041	0.968	0.978	0.032	0.022
	4	0.893	0.827	0.107	0.173	0.873	0.824	0.127	0.176	0.826	0.817	0.174	0.183
	8	0.889	0.893	0.111	0.107	0.888	0.894	0.112	0.106	0.907	0.892	0.093	0.108
	12	0.903	0.904	0.098	0.096	0.916	0.911	0.084	0.089	0.919	0.926	0.081	0.074
	16	0.908	0.905	0.092	0.095	0.907	0.913	0.093	0.087	0.943	0.943	0.057	0.057
O. sativa	20	0.905	0.904	0.095	0.096	0.912	0.912	0.088	0.088	0.948	0.949	0.052	0.051
(Rice)	24	0.907	0.901	0.093	0.099	0.924	0.912	0.076	0.088	0.945	0.950	0.055	0.050
	28	0.907	0.901	0.093	0.099	0.919	0.911	0.081	0.089	0.946	0.950	0.054	0.050
	32	0.905	0.899	0.096	0.101	0.921	0.910	0.080	0.090	0.952	0.950	0.049	0.050
	36	0.905	0.899	0.095	0.101	0.922	0.908	0.078	0.092	0.948	0.950	0.052	0.050
	40	0.906	0.897	0.094	0.103	0.922	0.907	0.078	0.093	0.948	0.949	0.052	0.051

[a] The percentage of composite and non-composite clusters calculated directly from simulations of *de novo* read clustering.

[b] The percentage of composite ($1-a_1$) and non-composite (a_1) clusters estimated by EM approximation of parameters in the mixed Poisson model.
