# Supplementary material for: Reference-free SNP calling: improved accuracy by preventing incorrect calls from repetitive genomic regions
Source: Biol Direct. 2012 Jun 8;7:17. doi: 10.1186/1745-6150-7-17 (PMC3472322; doi:10.1186/1745-6150-7-17)
Supplement: Additional file 3 — Table S3. False positive rates and false negative rates calculated for four SNP calling approaches based on the simulation datasets of Arabidopsis thaliana and Oryza sativa. [file 1745-6150-7-17-S3.pdf]

Table S3. False positive rates (FPRs) calculated for four SNP calling approaches based on the simulation datasets of *Arabidopsis thaliana* and *Oryza sativa*.

Read length	Cov[a]	*A. thaliana*				*O. sativa*			
		iML_denovo[b]	ML_denovo[c]	TH_denovo[d]	ML_ref[e]	iML_denovo	ML_denovo	TH_denovo	ML_ref
35bp	4	0.5566	0.6693	0.4132	0.1260	0.6271	0.7441	0.5519	0.1311
	8	0.1774	0.3026	0.1871	0.1301	0.2623	0.4300	0.2973	0.1258
	12	0.0983	0.2206	0.1675	0.0919	0.1446	0.3284	0.2518	0.0866
	16	0.0745	0.1997	0.1563	0.0333	0.1212	0.3079	0.2354	0.0350
	20	0.0458	0.1943	0.1451	0.0169	0.0815	0.3048	0.2215	0.0129
	24	0.0415	0.2024	0.1427	0.0062	0.0654	0.2992	0.2180	0.0063
	28	0.0286	0.1932	0.1382	0.0028	0.0536	0.2981	0.2135	0.0031
	32	0.0225	0.1901	0.1377	0.0010	0.0646	0.2971	0.2055	0.0030
	36	0.0115	0.1908	0.1317	0.0010	0.0584	0.2872	0.2070	0.0023
	40	0.0112	0.1963	0.1293	0.0003	0.0558	0.2865	0.2017	0.0016
50bp	4	0.4631	0.6111	0.2882	0.1278	0.5856	0.7238	0.4255	1.0000
	8	0.1298	0.2247	0.1178	0.1224	0.2190	0.3562	0.2050	0.1307
	12	0.0624	0.1446	0.0944	0.0814	0.1099	0.2486	0.1705	0.0864
	16	0.0457	0.1266	0.0863	0.0363	0.0870	0.2221	0.1574	0.0370
	20	0.0318	0.1239	0.0854	0.0104	0.0608	0.2169	0.1511	0.0135
	24	0.0240	0.1209	0.0804	0.0056	0.0512	0.2188	0.1449	0.0079
	28	0.0213	0.1212	0.0804	0.0016	0.0425	0.2160	0.1442	0.0040
	32	0.0156	0.1223	0.0770	0.0009	0.0350	0.2206	0.1365	0.0018
	36	0.0128	0.1205	0.0748	0.0006	0.0296	0.2188	0.1346	0.0007
	40	0.0104	0.1213	0.0724	0.0004	0.0237	0.2195	0.1338	0.0011
100bp	4	0.3214	0.4976	0.1190	0.1072	0.4402	0.6466	0.2167	0.1143
	8	0.0967	0.1623	0.0436	0.1110	0.1788	0.2879	0.0842	0.1065
	12	0.0427	0.0851	0.0349	0.0819	0.0915	0.1685	0.0709	0.0811
	16	0.0350	0.0678	0.0317	0.0394	0.0581	0.1211	0.0624	0.0411
	20	0.0214	0.0563	0.0288	0.0169	0.0375	0.1051	0.0600	0.0411
	24	0.0143	0.0498	0.0281	0.0070	0.0297	0.0972	0.0583	0.0070
	28	0.0118	0.0482	0.0268	0.0028	0.0258	0.0974	0.0544	0.0070
	32	0.0102	0.0492	0.0270	0.0016	0.0214	0.0977	0.0537	0.0020
	36	0.0120	0.0521	0.0252	0.0007	0.0199	0.0987	0.0522	0.0013
	40	0.0085	0.0509	0.0250	0.0007	0.0177	0.0991	0.0524	0.0011

[a] Coverage of the simulated sequencing;

[b] iML_denovo, *de novo* SNP calling using the iML algorithm.

[c] ML_denovo, *de novo* SNP calling using the ML algorithm;

[d] TH_denovo, *de novo* SNP calling using the threshold approach;

[e] ML_ref, reference-based SNP calling using the ML algorithm;

Table S4. False negative rates (FNRs) calculated for four SNP calling approaches based on the simulation datasets of *Arabidopsis thaliana* and *Oryza sativa*.

Read length	Cov[a]	*A. thaliana*				*O. sativa*			
		iML_denovo[b]	ML_denovo[c]	TH_denovo[d]	ML_ref[e]	iML_denovo	ML_denovo	TH_denovo	ML_ref
35bp	4	0.9172	0.9140	0.8324	0.8148	0.9131	0.9070	0.8620	0.8001
	8	0.5330	0.5296	0.5413	0.3486	0.5753	0.5679	0.6226	0.3222
	12	0.2663	0.2606	0.4093	0.1348	0.3220	0.3128	0.5158	0.1033
	16	0.1525	0.1472	0.3395	0.0491	0.2380	0.2282	0.4588	0.0236
	20	0.1203	0.1119	0.2991	0.0304	0.1985	0.1847	0.4138	0.0026
	24	0.1076	0.0989	0.2661	0.0251	0.1877	0.1725	0.4013	0.0057
	28	0.0929	0.0840	0.2450	0.0220	0.1866	0.1699	0.3795	0.0015
	32	0.0932	0.0823	0.2243	0.0221	0.1831	0.1447	0.3586	0.0039
	36	0.0956	0.0843	0.2048	0.0235	0.1921	0.1419	0.3470	0.0020
	40	0.0955	0.0838	0.1909	0.0257	0.1839	0.1238	0.3343	0.0010
50bp	4	0.9391	0.9375	0.8275	0.8406	0.9384	0.9349	0.8499	1.0000
	8	0.5775	0.5740	0.5369	0.3802	0.6105	0.6049	0.6022	0.3862
	12	0.2820	0.2783	0.4047	0.1459	0.3446	0.3378	0.4891	0.1636
	16	0.1656	0.1602	0.3303	0.0723	0.2349	0.2264	0.4274	0.0866
	20	0.1205	0.1145	0.2882	0.0449	0.1990	0.1887	0.3923	0.0473
	24	0.1004	0.0934	0.2517	0.0361	0.1866	0.1760	0.3670	0.0590
	28	0.0970	0.0883	0.2296	0.0339	0.1741	0.1622	0.3463	0.0229
	32	0.0947	0.0849	0.2066	0.0309	0.1820	0.1684	0.3240	0.0644
	36	0.0899	0.0820	0.1874	0.0287	0.1737	0.1602	0.3131	0.0535
	40	0.0904	0.0823	0.1798	0.0299	0.1751	0.1606	0.2994	0.0471
100bp	4	0.9805	0.9796	0.9719	0.9069	0.9788	0.9776	0.9893	0.9030
	8	0.7910	0.7891	0.7859	0.5584	0.7972	0.7946	0.7773	0.5530
	12	0.5238	0.5206	0.5354	0.3129	0.5423	0.5380	0.5657	0.3019
	16	0.3205	0.3166	0.3961	0.2010	0.3528	0.3481	0.4279	0.2046
	20	0.2073	0.2024	0.2973	0.1608	0.2468	0.2403	0.3578	0.1959
	24	0.1428	0.1376	0.2686	0.1332	0.1996	0.1926	0.3368	0.1295
	28	0.1278	0.1220	0.2434	0.1235	0.1837	0.1762	0.3098	0.1340
	32	0.1224	0.1159	0.2264	0.1123	0.1772	0.1683	0.2928	0.1112
	36	0.1277	0.1213	0.2059	0.1114	0.1752	0.1666	0.2750	0.1067
	40	0.1188	0.1123	0.1974	0.1107	0.1738	0.1657	0.2640	0.1066

[a] Coverage of the simulated sequencing;

[b] iML_denovo, *de novo* SNP calling using the iML algorithm.

[c] ML_denovo, *de novo* SNP calling using the ML algorithm;

[d] TH_denovo, *de novo* SNP calling using the threshold approach;

[e] ML_ref, reference-based SNP calling using the ML algorithm;
